# HvHMA2, a P_1B_-ATPase from Barley, Is Highly Conserved among Cereals and Functions in Zn and Cd Transport

**DOI:** 10.1371/journal.pone.0042640

**Published:** 2012-08-03

**Authors:** Rebecca F. Mills, Kerry A. Peaston, John Runions, Lorraine E. Williams

**Affiliations:** 1 Centre for Biological Sciences, University of Southampton, Highfield Campus, Southampton, United Kingdom; 2 Oxford Brookes University, School of Life Sciences, Oxford, United Kingdom; Kansas State University, United States of America

## Abstract

Manipulation of crops to improve their nutritional value (biofortification) and optimisation of plants for removal of toxic metals from contaminated soils (phytoremediation) are major goals. Identification of membrane transporters with roles in zinc and cadmium transport would be useful for both aspects. The P_1B_-ATPases play important roles in heavy metal allocation and detoxification in Arabidopsis and it is now important to elucidate their roles in monocots. We identified nine P_1B_-ATPases in barley and this study focuses on the functional characterization of HvHMA2, providing evidence for its role in heavy metal transport. *HvHMA2* was cloned using information from EST analysis and 5′ RACE. It possesses the conserved aspartate that is phosphorylated during the reaction cycle of P-type pumps and has motifs and key residues characteristic of P_1B_-ATPases, falling into the P_1B-2_ subclass. Homologous sequences occur in three major sub-families of the Poaceae (Gramineae). Heterologous expression in *Saccharomyces cerevisiae* demonstrates that HvHMA2 functions as a Zn and Cd pump. Mutagenesis studies show that proposed cation coordination sites of the P_1B-2_ pumps are crucial for the metal responses conferred by HvHMA2 in yeast. *HvHMA2* expression suppresses the Zn-deficient phenotype of the Arabidopsis *hma2hma4* mutant indicating that HvHMA2 functions as a Zn pump *in planta* and could play a role in root to shoot Zn transport. When expressed in Arabidopsis, HvHMA2 localises predominantly to the plasma membrane.

## Introduction

Plants require a range of metals in trace amounts for growth and development. These metal micronutrients include Fe, Cu, Co, Zn, Mn and Ni [1]. They can play critical structural roles in many proteins; act as catalytic components in enzymes, and function in redox reactions. The correct balance of these micronutrients is required for optimum growth and development and complex mechanisms have evolved to ensure that proteins are supplied with adequate levels of the required metal and also to cope with fluctuations in the environment [2], [3].

Zn is required for all organisms, including plants. Zn deficiency is one of the most common micronutrient deficiencies in agricultural soils and thus can lead to reductions in crop yield. Zn is also essential in human nutrition and Zn deficiency is estimated to affect more than 25% of the world's population causing impaired growth and increased susceptibility to disease. Plants at the base of the food chain are an important source of dietary Zn. Zn tends to be at a relatively low level in staple foods, and cereals such as barley, wheat and rice have relatively low levels in the grain. Therefore Zn biofortification of food crops which could lead to increased bioavailable Zn would be an important sustainable solution to address Zn malnutrition [4]. Understanding the processes that contribute to Zn uptake from the soil, translocation to the shoot and partitioning in the grain are integral to developing strategies to improve the Zn content of grain. Cadmium is a non-essential metal that can contaminate soils and is toxic to both plants and animals. It can be taken up by transporters of essential micronutrients such as Zn and Fe [5]; therefore when considering mechanisms to increase the Zn content of food it is also necessary to consider their potential to accumulate Cd [4].

The P_1B_-ATPases (also known as Heavy Metal ATPases or HMAs) are one of several transporter families involved in Zn transport [6], [7]. P_1B_-ATPases are classified into six subgroups (P_1B1-6_) which are proposed to have distinct metal binding and transport specificities [8]. There are eight P_1B_-ATPases in *Arabidopsis thaliana* and four have some role in Zn transport. AtHMA1 is found at the inner envelope of the chloroplast and contributes to Zn(II) detoxification by reducing the Zn content of plastids [9]. It is also reported to load Cu into the stroma, supply Cu to chloroplast Cu/Zn superoxide dismutase [10], and transport Ca [11]. AtHMA2 and AtHMA4 play key roles in translocation of Zn from root to shoot with the *hma2hma4* double mutant exhibiting a strong Zn nutritional deficiency [12], [13]. They are also the main route by which Cd is transferred to the shoot [14]. AtHMA4 also plays a role in Cd detoxification at elevated Cd levels [15]. AtHMA3 is proposed to function in vacuolar sequestration of Zn, Cd, Co and Pb, suggesting a detoxification role [16].

As many of our staple food sources such as the cereals rice and wheat are monocots, it is important to understand the function of P_1B_-ATPases in these species. From genome sequence analysis, nine HMA genes have been identified in rice (OsHMA1-9). The first to be characterised was OsHMA9 [17]. Phylogenetically OsHMA9 clusters with the Arabidopsis Cu pumps AtHMA5-8 [18]; however, phenotypic analysis of rice *oshma9* mutants suggested a broader role as mutants were sensitive to high Cu, Zn, Cd and Pb [17]. Subsequently OsHMA3, which clusters with AtHMA2, 3 and 4 in the Zn/Cd/Pb (P_1B-2_) sub-group, was shown to be a vacuolar Cd uptake transporter in roots, reducing cytoplasmic Cd levels and consequently transport of Cd to the shoot [19], [20], [21]. It remains to be shown whether OsHMA3 also transports other metals. Recently OsHMA2 was shown to mediate Cd efflux when expressed in yeast [22] and mutant analysis in rice suggests that OsHMA2 is a major transporter of Zn and Cd from roots to shoots [23]. Despite the importance of this family of transporters, we know virtually nothing in the major temperate cereals such as wheat and barley. To address this we describe the cloning and functional analysis of HvHMA2, a barley P_1B_-ATPase from the Zn/Cd/Pb (P_1B-2_) sub-group. Using a variety of approaches we show that it can transport the essential micronutrient Zn; however it can also transport the toxic contaminant Cd. We also show that key residues postulated to form part of the cation-binding site in P_1B-2_-ATPases are crucial for the metal responses conferred by HvHMA2 in yeast.

## Results

### Primary structure of HvHMA2

**Figure 1:**
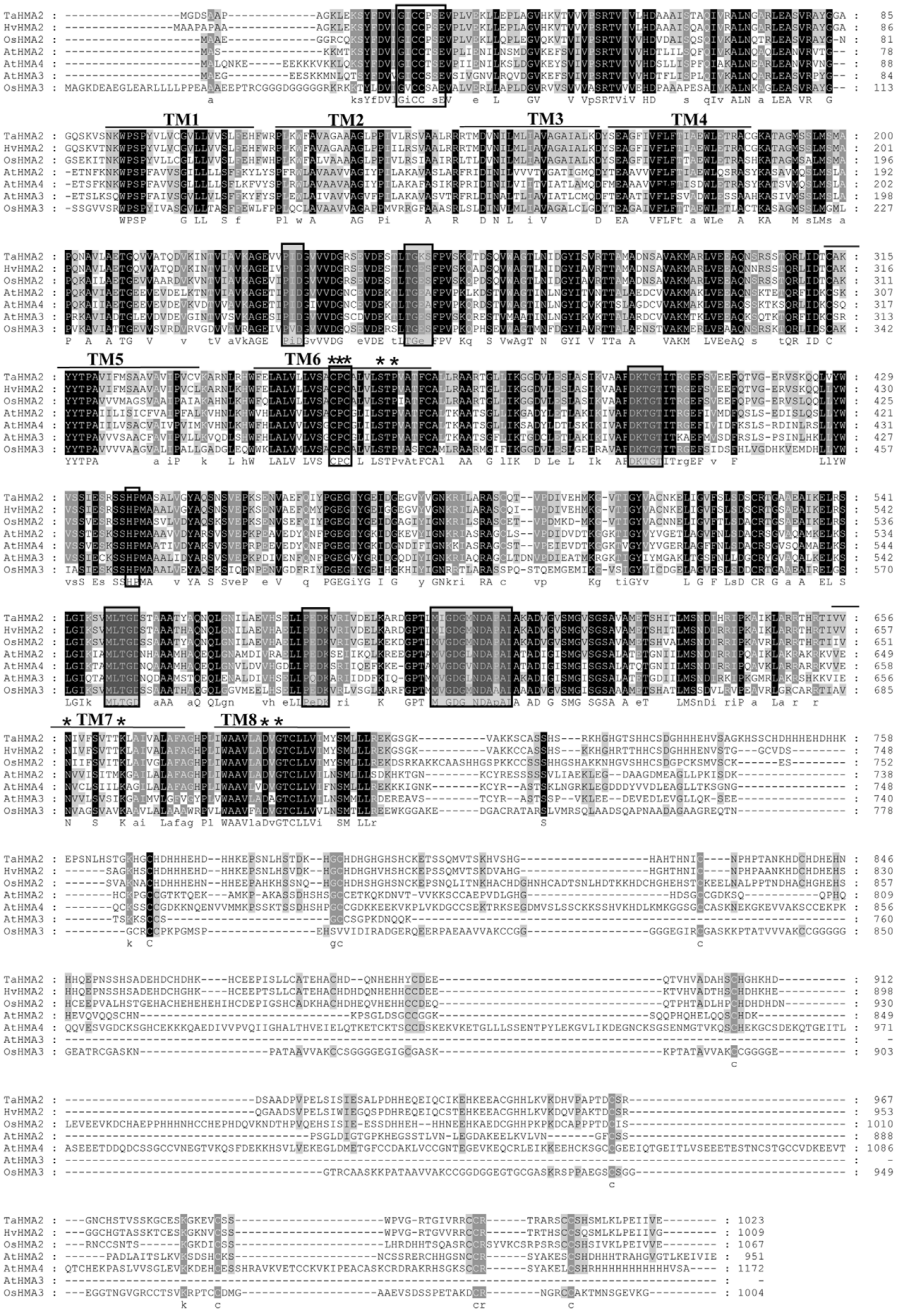
Alignment of HvHMA2 with various plant P_1B_-ATPases showing the predicted TMs. Swissprot TM predictions for AtHMA2 are indicated, except TM6 was extended according to the SOSUI prediction. Shaded boxes indicate motifs conserved in P-type ATPases, other boxes indicate motifs in P_1B_-ATPase subgroup, and asterisks indicate residues conserved in P_1B-2_-ATPases that may co-ordinate the metal ion during transmembrane transport and contribute to ion specificity. Conserved (upper-case) and semi-conserved (lower-case) amino acids are indicated beneath the alignment.

HvHMA2 was amplified by RT-PCR using sequence information from EST analysis and 5′ RACE (figure S1). HvHMA2 contains an open reading frame of 3027 bp, encoding a protein of 1009 amino acids and 108,456 molecular mass. Proteins showing high similarity in the P_1B-2_ subclass include putative heavy-metal transporting P-type ATPases from plants and bacteria (http://www.ncbi.nlm.nih.gov/BLAST/). Highest homology (91% identity) is to a wheat sequence TaHMA2, which has not yet been functionally characterized. Full-length homologues of HvHMA2 were also identified in other members of the Poaceae: two in brachypodium and rice, three in sorghum and four in maize. A barley OsHMA3 homologue (HvHMA3) was also recently submitted to NCBI and has 52% identity to HvHMA2. Percentage identities and alignments of HvHMA2 to homologues in other plants are shown in Table S1 and Figure 1. Hydropathy analysis suggests HvHMA2 has 8 transmembrane domains (TMs) (figure S2).

**Figure 2:**
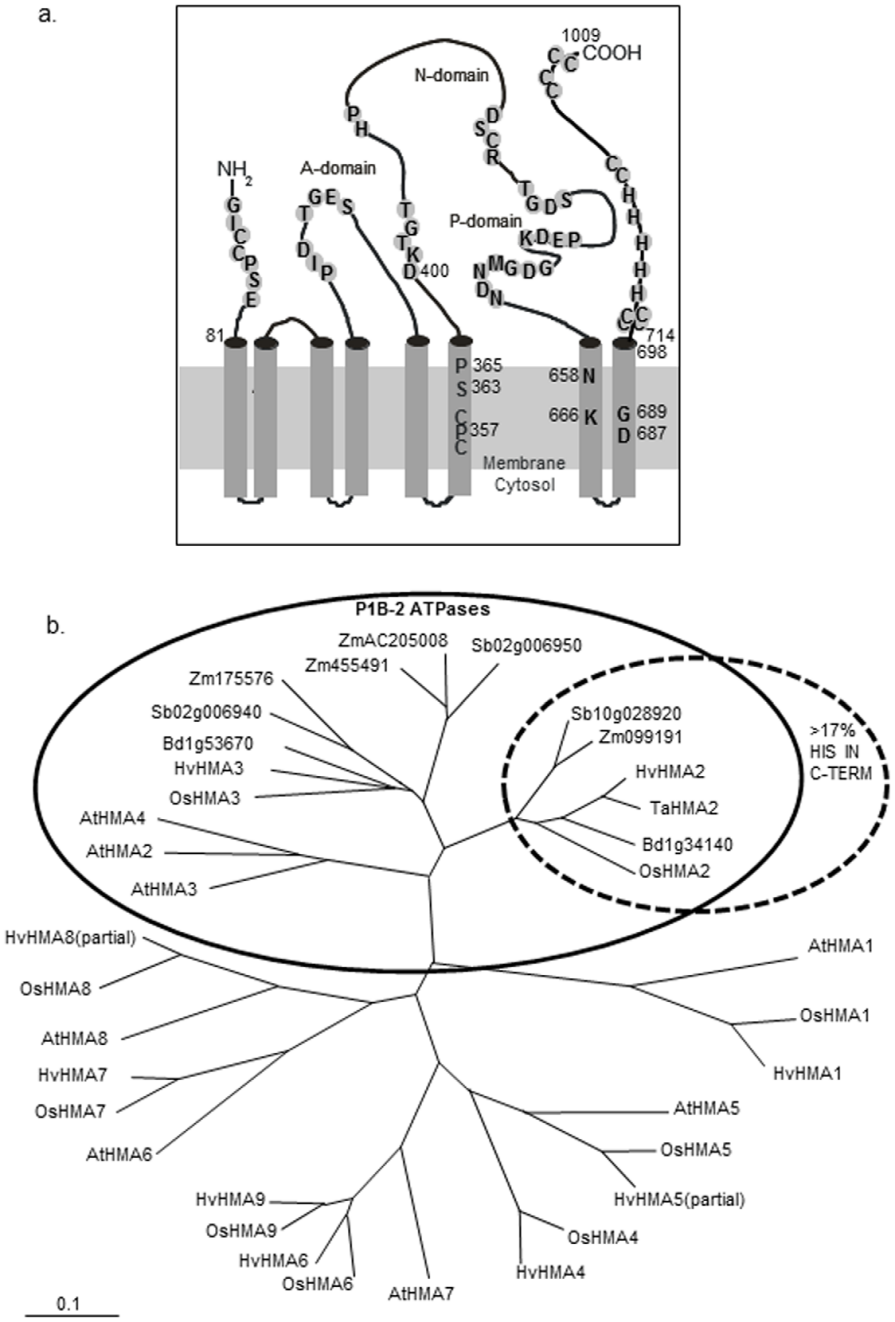
a. Prediction model for the transmembrane topology of HvHMA2. Schematic diagram illustrating predicted TMs and key motifs. Residues shown in TMs are postulated to coordinate metals during transport. For HvHMA2 the putative cytoplasmic C-terminal metal-binding domain is 307 aa including 56 His and 18 Cys residues plus 4 Cys pairs. Amino acid numbers relevant to the deletion and substitution mutants are included. b. Dendrogram of P_1B_-ATPases. Includes: all rice and Arabidopsis P_1B_-ATPases, best available barley sequences and predicted P_1B-2_-ATPases identified through Aramemnon for maize, Brachypodium and sorghum. The P_1B-2_ (Zn/Cd) ATPase subgroup is circled, and the sub-set with >17% His residues in the predicted C-termini is indicated. Those P_1B-2_-ATPases that do not fall within this subset feature <9% His residues in the predicted C-termini. Partial sequences are indicated. Scale bar indicates amino acid substitutions per site.

HvHMA2 and the wheat homologue TaHMA2 contain motifs found in all P-type ATPases including the conserved aspartate (D_400_ in HvHMA2, D_399_ in TaHMA2) that is phosphorylated during the reaction cycle (Figure 1 and 2a). Both also have motifs characteristic of P_1B_-ATPases [18] including the HP locus in the predicted large cytoplasmic loop (present in most P_1B_-ATPases but not in other P-types) (Figures 1 and 2a). P_1B_-ATPases usually have putative heavy Metal-Binding Domains (MBDs) in the N or C termini and the CPx/SPC motif in TM6 [18], [13]. HvHMA2 and TaHMA2 contain the CPC motif in the predicted TM6 (C_356_ PC in HvHMA2). A “heavy-metal-associated domain” in the HvHMA2 and TaHMA2 N-termini is recognized by the pfam and PROSITE databases (http://ca.expasy.org/prosite; www.sanger.ac.uk/software/pfam). Within this domain the motif GxCCxxE occurs in all the plant P_1B-2_ sub-class, whereas one or more copies of the motif GMxCxxC occur in P_1B-2_ ATPases from other organisms.

P_IB_-ATPases are classified into subsets 1–6 depending on potential cation coordinating residues present in the 6^th^, 7^th^ and 8^th^ TMs [8], modified by [18]. For the P_1B-2_ Zn/Cd/Pb transporting sub-group these are TM6: CPCx_4_SxP; TM7: Nx_7_K; TM8: DxG (shown for HvHMA2 in Figure 2a, Table S2). Numbered for HvHMA2 (subtract 1 for TaHMA2 numbers) they are: C356, P357, C358, S363 and P365 in TM6, N658 and K666 in TM7 and D687 and G689 in TM8 (Figure 1, Figure 2a).

### Phylogenetic analysis of barley HMAs

The phylogenetic tree (Figure 2b) relates HvHMA2 to rice P_1B_-type ATPase sequences, their barley homologues, Arabidopsis P_1B_-types, and HvHMA2 (P_1B-2_) homologues from other monocots. Separate sequences previously classified as distinct HMAs, HvHMA1 and HvHMA10, are here combined as HvHMA1 in line with rice genes and recent barley EST data.

The monocot P_1B-2_ ATPases identified here fall into two subgroups that differ notably in the composition of their predicted cytoplasmic C-termini; those of HvHMA2, TaHMA2, OsHMA2, maize, sorghum and brachypodium homologues contain high percentages of His residues (>17%) as well as up to 6 CC pairs distributed throughout (Figures 1 and 2b). Those that lack the His-rich C-termini (including OsHMA3 and HvHMA3) have a conserved W instead of the conserved G between TM7 and TM8 (G_676_ in HvHMA2).

### Functional analysis of HvHMA2 in *Saccharomyces cerevisiae*

#### HvHMA2 transports Zn and Cd in yeast

**Figure 3:**
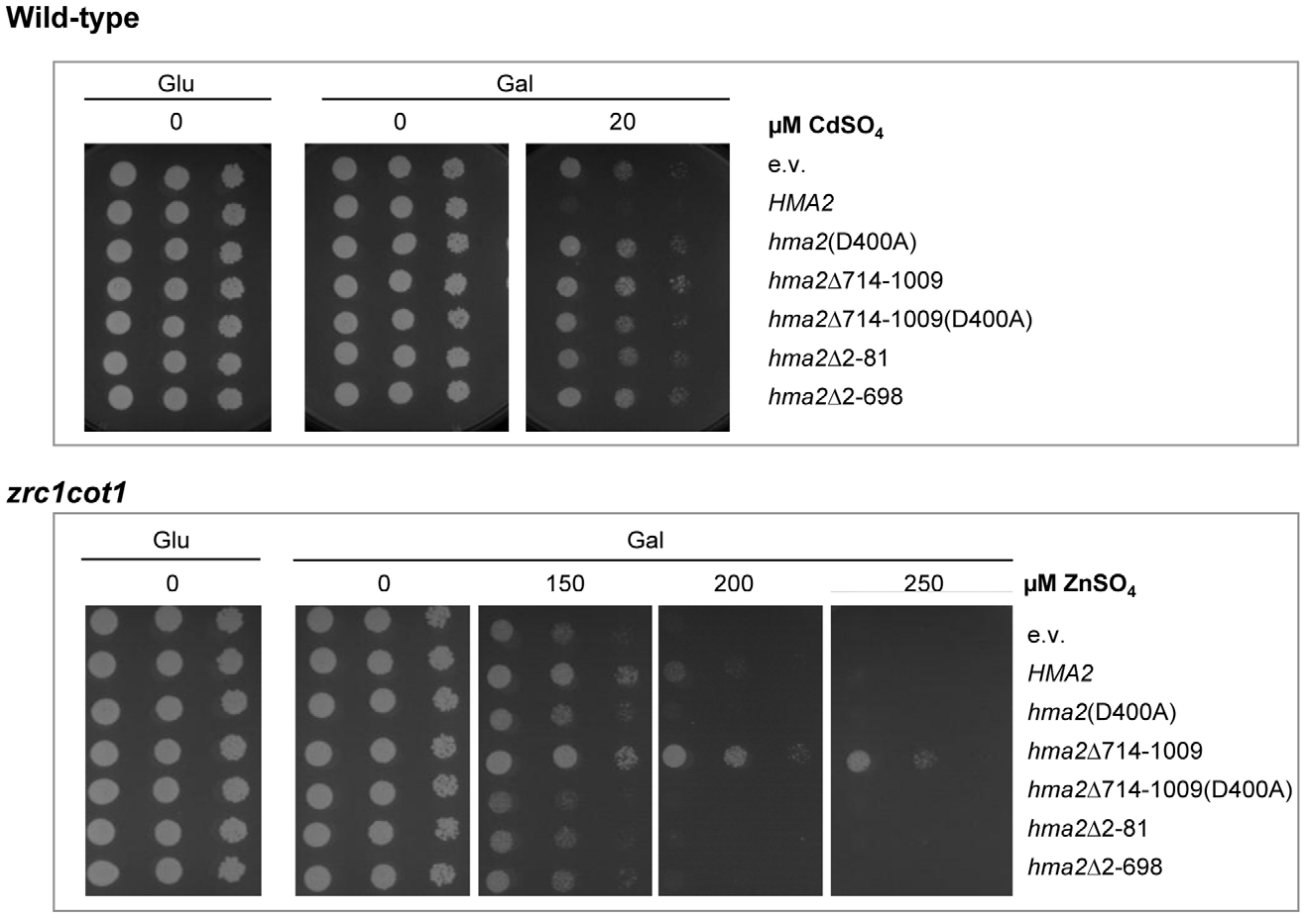
Heterologous expression of HvHMA2 in yeast. HvHMA2 expression under a Gal-inducible promoter confers Cd sensitivity to wild-type yeast (top) and Zn resistance to *zrc1cot1* mutant yeast (bottom) compared to empty vector (e.v) transformed control yeast. Mutant forms of the pump are: C-terminally deleted, hma2Δ714-1009; N-terminally deleted, hma2Δ2-81; C-terminal region alone, hma2Δ2-698; mutant with critical aspartate mutated, hma2(D400A). Photographs show undiluted, 1/10 and 1/100 dilutions of aliquots on agar containing either glucose or galactose as the carbon source, and varying concentrations of CdSO_4_ or ZnSO_4_ as indicated.

**Figure 4:**
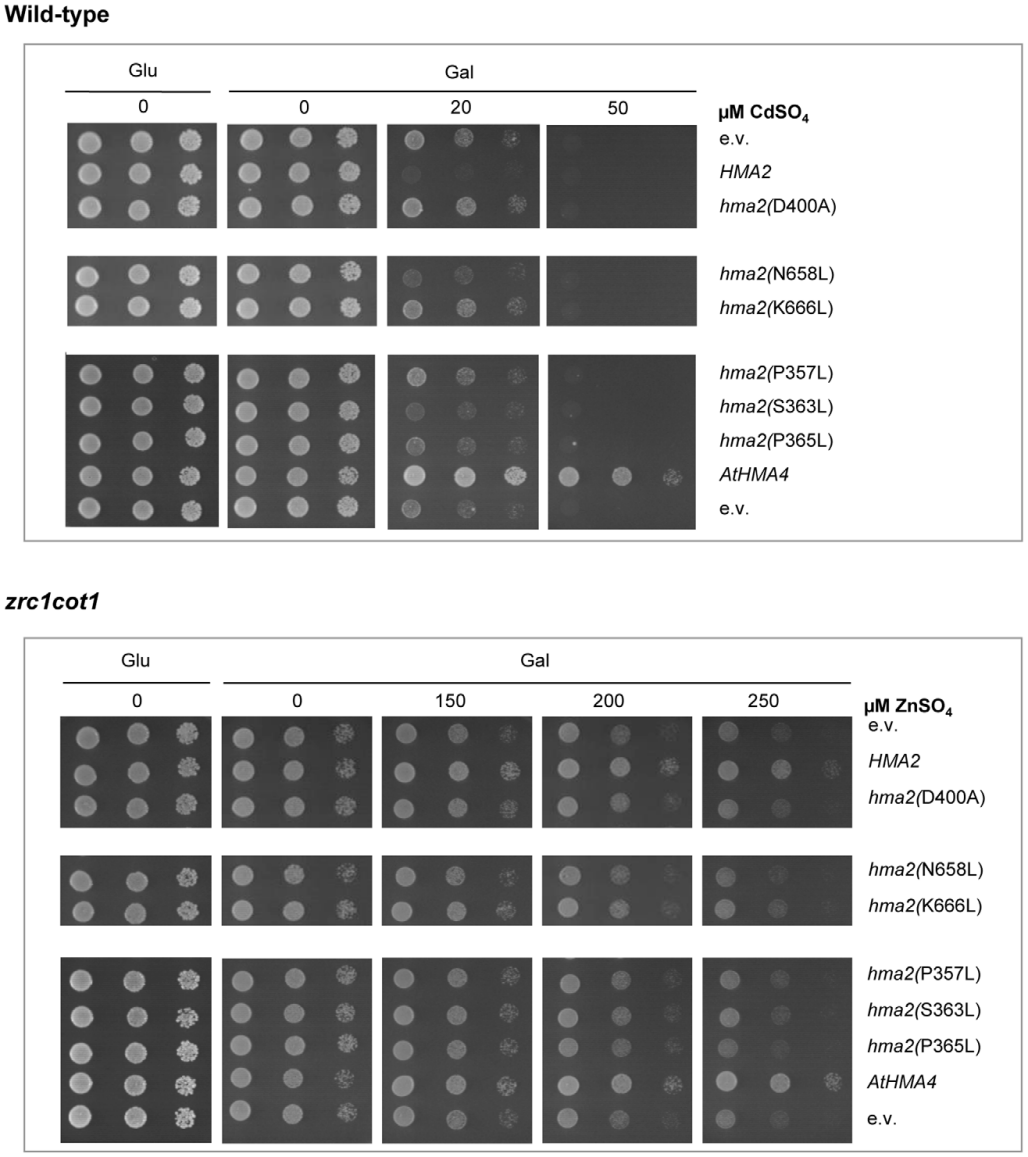
Mutating key residues in HvHMA2 alters metal responses. Mutation of predicted critical residues decreases the Cd sensitivity conferred by HvHMA2 on wt yeast (top) and the Zn resistance conferred on *zrc1cot1* mutant yeast (bottom). Yeast are transformed with galactose-inducible HvHMA2, HvHMA2 with mutation of N658, S363, P365, K666 or P357 to leucine or D400 to alanine, AtHMA4 or empty vector (e.v). Photographs show undiluted, 1/10 and 1/100 dilutions of aliquots on agar containing either glucose or galactose as the carbon source, with CdSO_4_ or ZnSO_4_ concentrations as indicated.

HvHMA2 conferred Cd sensitivity to wild-type (wt) yeast (Figure 3). A transport deficient mutant was produced in which the conserved aspartate residue of HvHMA2 was mutated to alanine, *hvhma2(D400A)*. Cd sensitivity was abolished in this mutant, indicating that HvHMA2-dependent Cd sensitivity was due to transport activity. A similar response was also seen in the Cd^2+^-sensitive *ycf1* yeast mutant (results not shown). Previously we have shown that AtHMA4 confers Cd resistance to wt yeast when expressed in the p426 vector [15], [13]. To demonstrate that the effects observed were not due to different vectors, we expressed AtHMA4 in the pYTV vector used in this study for HvHMA2. AtHMA4 still confers resistance to Cd in this vector (Figure 4).

Expression of HvHMA2 partially alleviated the Zn sensitivity of the *zrc1cot1* mutant (Figure 3) and this effect was lost in the D400A mutant indicating that HvHMA2 can transport Zn. HvHMA2 had no marked effect on the Cu, Co, Ni or Mn sensitivity of wt yeast (data not shown).

#### Role of HvHMA2 N and C termini

The C-terminally truncated version (HvHMA2Δ714-1009) which has the last 296 residues deleted confers marked Zn resistance on the *zrc1cot1* mutant; this is due to transport function as it is eliminated in the corresponding D400A mutant (HvHMA2Δ714-1009(D400A)) (Figure 3). C-terminal truncation of HvHMA2 completely abolished its ability to confer Cd sensitivity (Figure 3). This truncated version, like the full-length HvHMA2, had no effect on the Cu, Co, Ni or Mn sensitivity of wt yeast (data not shown). Deletion of the N-terminus from HvHMA2 (HvHMA2Δ2-81) eliminated Cd and Zn transport (Figure 3). No effect of expression of HvHMA2Δ2-81 was observed on Cu, Co, or Ni sensitivity of wt yeast (data not shown). Expression of just the C-terminus part of HvHMA2 (HvHMA2Δ2-698) has no marked effect on Cd sensitivity in wt yeast, Zn sensitivity of *zrc1cot1* (Figure 3), or on Cu, Co, Ni or Mn sensitivity of wt yeast (data not shown).

#### Effect of mutations in putative metal coordination sites in HvHMA2

To investigate the functional significance of some of the invariant residues in TM6 and 7 for P_1B-2_ ATPases (see above) we generated the HvHMA2 mutants, P357L, S363L, P365L, N658L and K666L, and expressed them in yeast. The HvHMA2 mutation P357L alters a predicted critical residue/ion specificity determinant in TM6. This proline is part of the CPC motif, a characteristic motif found in P_1B_-ATPases. As seen in Figure 4, the HvHMA2(P357L) mutant no longer conferred Cd sensitivity to wt yeast and restored growth to control (e.v.) levels. It also abolished the slight Zn resistance conferred on *zrc1cot1* yeast mutant by HvHMA2. Two TM6 mutations, HvHMA2(S363L) and HvHMA2(P365L), also decreased the ability of HvHMA2 to confer Cd sensitivity to wt yeast and Zn resistance to *zrc1cot1* mutant yeast, although not quite to the same extent as the D400A or P357L mutations (Figure 4). The TM7 mutant HvHMA2(N658L) reduced but did not eliminate the Cd sensitivity conferred to wt yeast compared to the HvHMA2 construct whereas Cd sensitivity was abolished in the (TM7) HvHMA2(K666L) mutant (Figure 4). In the *zrc1cot1* yeast mutant both the N658L and K666L mutations abolished the Zn resistance conferred by HvHMA2 (Figure 4).

### Tissue and membrane distribution of HvHMA2

**Figure 5:**
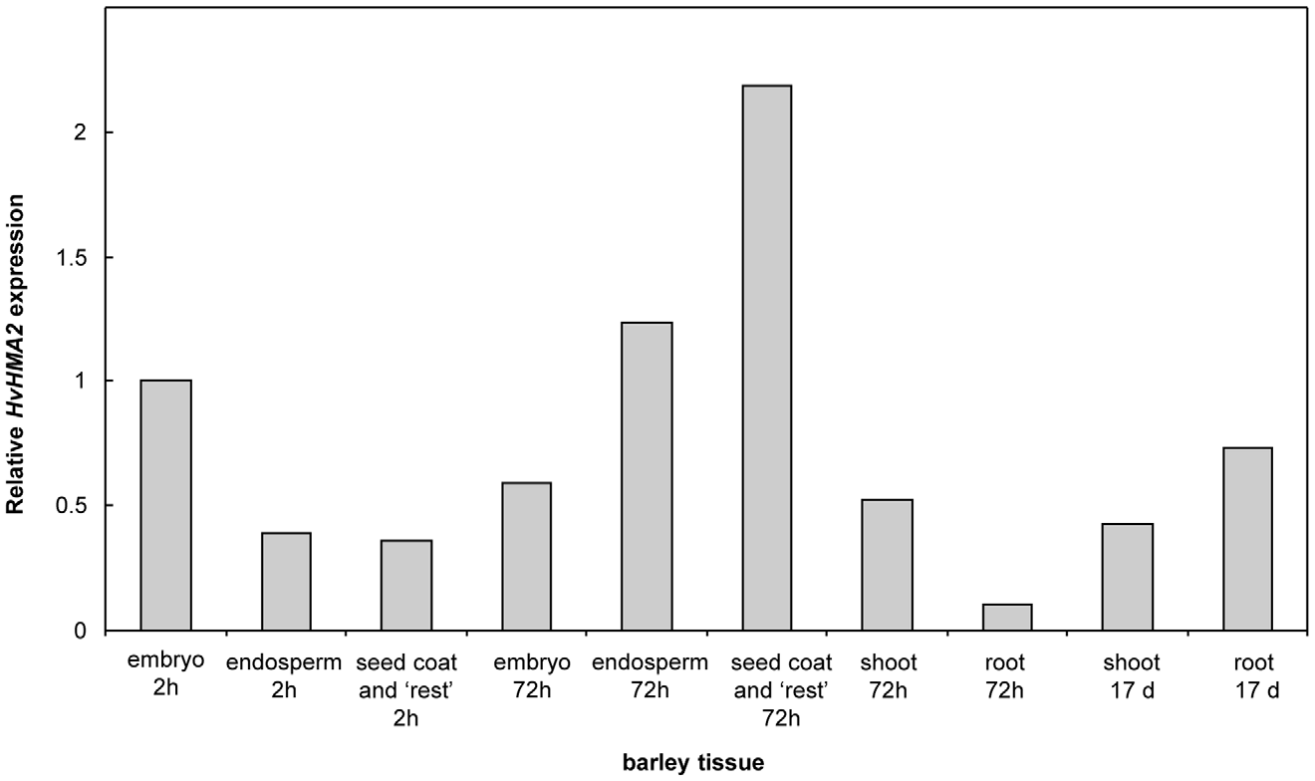
Expression pattern of HvHMA2 in different barley tissues. Relative HvHMA2 gene expression levels determined using real-time PCR (average of 2 biological repeats each repeated in triplicate). Time after imbibition, tissues as illustrated in supplementary figure 4.

**Figure 6:**
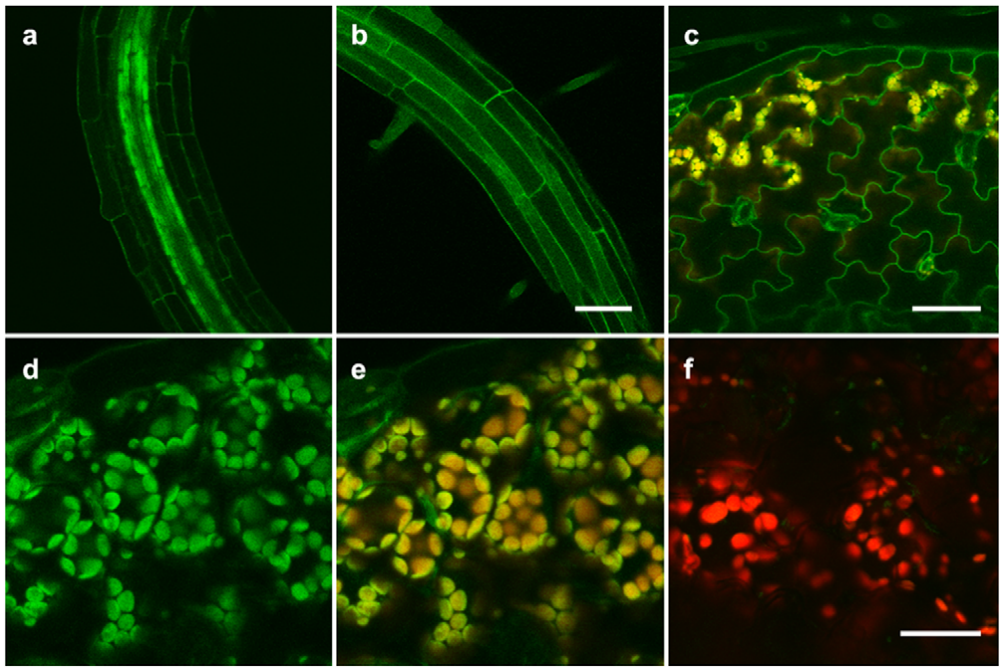
GFP-HvHMA2 localizes to the plasma membrane (PM) and chloroplasts of Arabidopsis. (a) The PM is marked in all root cells within the root-hair initiation zone. Cells of the stele appear very bright at this stage. (b) In more mature root regions, the PM is very bright. Scale bar (a and b) = 50 µm. (c) In cotyledons, GFP-HvHMA2 marks the PM of epidermal cells (green) and the chloroplasts of mesophyll cells (orange). Orange colouring in chloroplasts results from overlay of the GFP and chlorophyll autofluorescence signals. Scale bar = 50 µm. (d–e) Higher magnification of chloroplast localization in cotyledons. GFP and chlorophyll autofluorescence are overlain in (e). (f) Chlorophyll autofluorescence (red) from cotyledon mesophyll cells of a untransformed control plant overlain with green channel emission collected as in (e). Note that very little green signal appears in wt chloroplasts. Scale bar (d–f) = 25 µm.

Analysis of microarray data sets shows that *HvHMA2* and *TaHMA2* have similar expression patterns (Figure S3). We used real-time PCR to show that *HvHMA2* expression occurs in all tissues of the germinating grain (Figure S4, Figure 5). *HvHMA2* expression decreases in embryo and increases in endosperm tissue between 2 and 72 h, while highest expression is seen in seed coat and other tissue remaining after embryo and endosperm removal (Figure 5, figure S4). HvHMA2 was also expressed in more mature tissues, being found in both root and shoot tissues of 17 day old plants (Figure 5). To determine the membrane localisation of HvHMA2, it was expressed with a GFP tag in Arabidopsis. This showed that HvHMA2 was predominantly localised in the plasma membrane (PM) in root and cotyledon cells (Figure 6 a, b); in addition in some cotyledon cells it was detected in the chloroplasts (Figure 6 c–f).

### *HvHMA2* expression rescues the Zn-dependent growth phenotype of the Arabidopsis *hma2hma4* mutant

**Figure 7:**
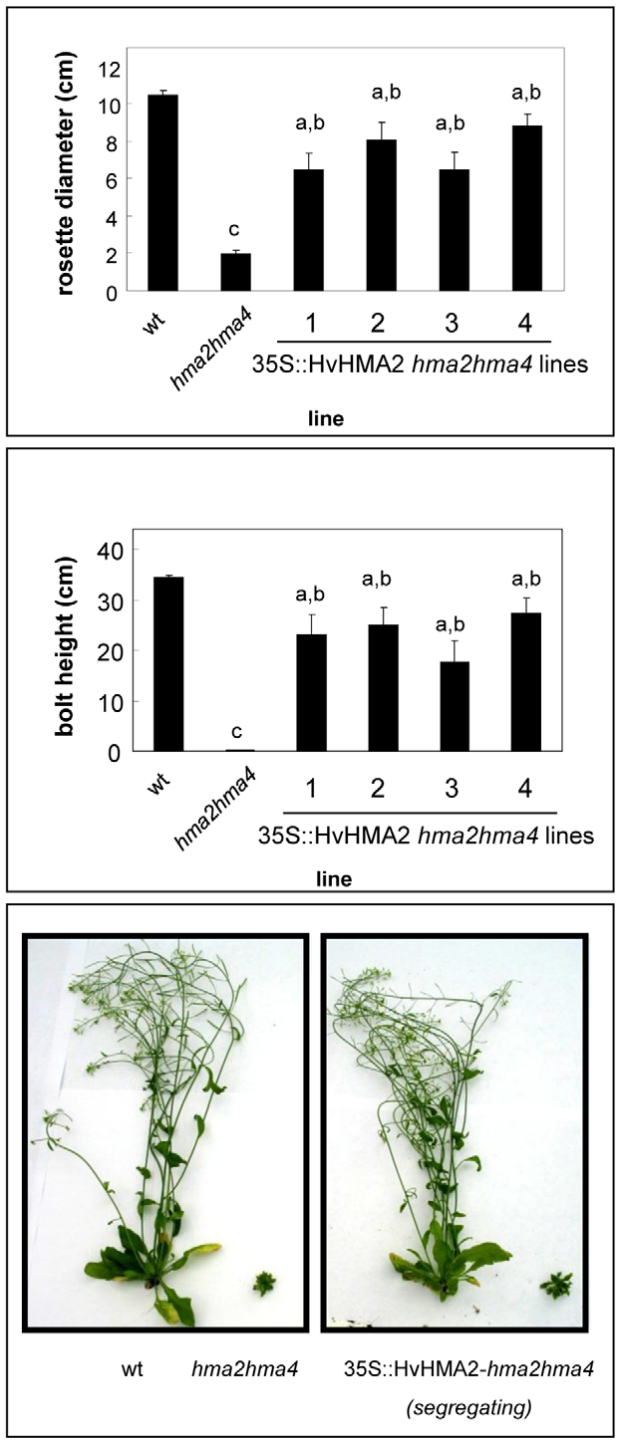
Expression of *HvHMA2* rescues the Zn-deficiency phenotype of the *hma2hma4* mutant. Growth of 35S::HvHMA2 *hma2hma4* plants compared to untransformed *hma2hma4* and wild-type (wt) plants. These were soil-grown plants not supplemented with nutrient solution. Top, rosette diameter and Middle, bolt height (41 days). Values are means+/−S.E (n = 12, T2 plants). Student's t-test was used to determine significance levels: a, significant difference between HvHMA2-expressing line and *hma2hma4* mutant (P<0.05); b, significant difference between HvHMA2-expressing line and wt (P<0.05); c, significant difference between wt and *hma2hma4* (P<0.05). Bottom, representative plants are shown.

**Figure 8:**
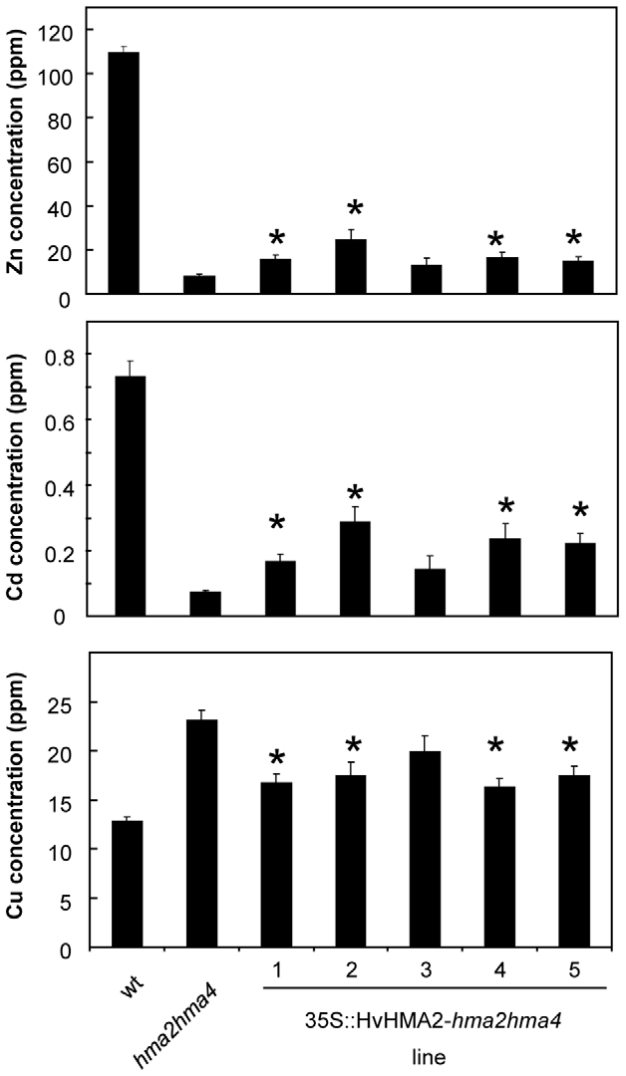
HvHMA2 partially restores the Zn, Cd and Cu shoot levels when expressed in *hma2hma4* mutants. Levels of Zn (top), Cd (middle) and Cu (bottom) in shoots of soil-grown plants. These were soil-grown plants supplemented with nutrient solution according to [42]. Wild-type (wt), *hma2hma4* mutant and 35S::HvHMA2-*hma2hma4* T2 plants are shown. Values are means+/−S.E (n = 12 plants). Student's t-test was used to determine significance levels: *, significant difference between HvHMA2-expressing line and *hma2hma4* mutant (P<0.05).

The Arabidopsis *hma2hma4* mutant is severely stunted due to the lack of Zn translocation from root to shoot, a process dependent on AtHMA2 and AtHMA4 [12]. This stunted phenotype was clearly seen under the conditions used in this study (Figure 7, figure S5) although we did not observe chlorosis [12]. The wt phenotype was restored to the mutant by supplying additional Zn to the plants [12; ] figure S5. To test whether HvHMA2 functions in Zn transport *in planta*, it was expressed in the *hma2hma4* mutant. Several independent lines were generated and expression of *HvHMA2* in these plants was confirmed using RT-PCR (figure S6). When grown on soil alongside wt and *hma2hma4* mutants, T2 plants of these lines segregated with an approximate 3∶1 distribution of wt∶stunted (*hma2hma4*-type) phenotype. PCR on genomic DNA isolated from T2 plants confirmed that *hma2hma4* mutant plants transformed with *HvHMA2* had a wt phenotype, whereas *HvHMA2* was not detected in those T2 transformant plants that had a stunted phenotype (data not shown). Suppression of the *hma2hma4* stunted phenotype by HvHMA2 is shown in Figure 7. Rosette diameter and bolt height were determined as a measure of rescue, and shows that these lines had a significantly greater average rosette diameter and bolt height than *hma2hma4* mutants (Figure 7). The T2 population was analysed because suppression of the stunted phenotype was markedly reduced in homozygous *HvHMA2-*transformant T3 plants, possibly due to silencing. To determine the effect of HvHMA2 expression on the ionomic profile, plants were grown on soil supplemented with Cd as well as essential micronutrients; under these conditions the Arabidopsis *hma2hma4* mutant has low shoot Zn and Cd concentrations and a high shoot Cu concentration compared to wt Arabidopsis (Figure 8; [13]). In *hma2hma4* mutant lines expressing *HvHMA2* (T2) the average shoot Zn, Cd and Cu levels were partially restored to the levels observed in wt Arabidopsis: Zn and Cd levels were increased compared to *hma2hma4*, while Cu levels were decreased (Figure 8).

### Silique growth is not fully rescued in the 35S::HvHMA2-*hma2hma4* lines and this correlates with reduced Zn content

**Figure 9:**
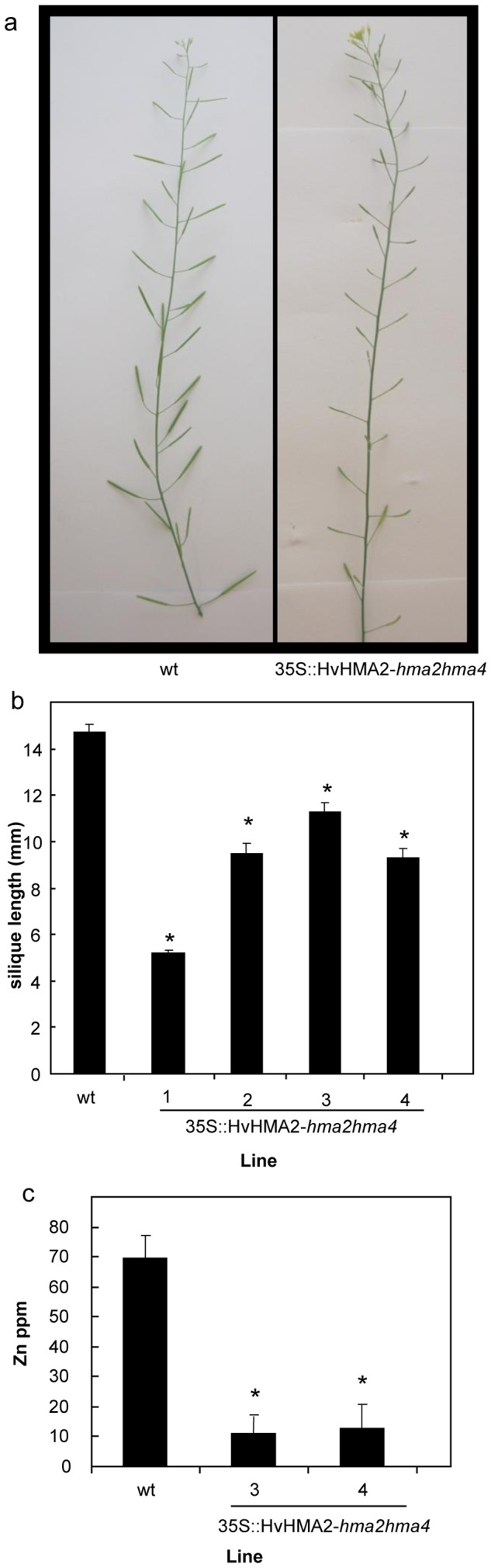
*HvHMA2*-expressing *hma2hma4* plants have shorter siliques than wild-type and are reduced in Zn content. (a). Images of siliques from wt and HvHMA2-expressing *hma2hma4* line. (b). Silique length. Values are means+/−S.E (n = 40 siliques from 5 plants producing bolts for each line). (c). Zn levels in siliques of wt and 35S::HvHMA2-*hma2hma4* lines. Results represent the mean Zn concentration determined from approximately 20 siliques per plant using three plants per line. For a, b and c plants were grown in soil for 49 days and had no additional nutrient supplementation. Student's t-test was used to determine significance levels: *, significant difference between transgenic lines and wt (P≤0.05).

When grown in soil with no nutrient supplementation, the *HvHMA2*-*hma2hma4* Arabidopsis plants flowered and formed siliques although these tended to be shorter than wt siliques (Figure 9a and b). This was also observed in *AtHMA4*-*hma2hma4* plants [13]. Viable seed were produced from these plants. Elemental analysis carried out on siliques showed that only Zn levels were significantly different in HvHMA2-*hma2hma4* plants compared to wt (Figure 9c).

## Discussion

Barley is a major crop and represents a good model for studying metal transport in cereals. We are focussing on the P_1B_-ATPase family of ion transporters to understand the role they play in metal transport and homeostasis. HMA2 homologues appear to be highly conserved in Poaceae and they all contain the residues that put them into the P_1B-2_ subclass [8]. Functional characterisation of Arabidopsis P_1B-2_ ATPases AtHMA2, 3 and 4 indicates they are Zn/Cd pumps [24], [15], [25], [26], [16]. AtHMA2 is at the PM of Arabidopsis root pericycle cells [12], [27], [28] while AtHMA3 is vacuolar [16]. Rice has two P_1B-2_ ATPases and evidence indicates OsHMA3 is a vacuolar Cd pump in roots (as yet there is no evidence that it can transport Zn; [19]) while OsHMA2 functions in Cd and Zn transport at the plasma membrane [23]. We provide evidence that barley possesses two P_1B-2_ ATPases, HvHMA2 and 3, and importantly we demonstrate that HvHMA2 is able to transport both Zn and Cd. Results indicate that HvHMA2 localises predominantly to the PM although we also detected some chloroplast expression. The PM localisation is consistent with data showing that HvHMA2 may function in root to shoot translocation of Zn while a function in the chloroplast would require further investigation.

### HvHMA2 functions as a Zn/Cd pump in yeast

Studies in yeast show that HvHMA2 can transport Zn and Cd. Heterologous expression in yeast has been used previously to indicate potential substrates for HMAs and there are reports of expression resulting in both metal sensitivity and resistance [24], [15], [29], [30], [13]. This probably relates to their predominant membrane location when expressed in yeast. For example, the ER is thought to be a Cd-sensitive compartment in yeast; if pumps expressed here transport Cd into the ER lumen then Cd sensitivity may be observed [30], [31], [32], [33]. PM expression could alternatively result in Cd resistance for an efflux pump. HvHMA2 confers Cd sensitivity to wt yeast and this is eliminated in a transport null mutant where the conserved aspartate necessary for phosphorylation and functioning of these pumps was mutated to alanine. This suggests that HvHMA2 is transporting Cd into a sensitive compartment. HvHMA2 can also function in Zn transport as indicated by the Zn resistance observed when expressing this pump in the Zn-sensitive *zrc1cot1* mutant. Similarly, this was eliminated in the transport null version of the pump. This suggests that a proportion of HvHMA2 may be expressed at the PM and function in efflux out of the cell and/or that transport of Zn into the compartment where HvHMA2 is localised confers Zn resistance to this yeast mutant. Dual locations for related pumps have been observed when expressed in yeast [33]. We were not able to observe a signal when HvHMA2-GFP was expressed in yeast, possibly due to low levels of expression or instability caused by the tag, and so we cannot comment on its localisation, but the ability of HvHMA2 to confer Cd sensitivity and Zn resistance in yeast facilitated further structure/function analysis. Future studies using epitope tags or an antibody to HvHMA2 could help elucidate the localisation of HvHMA2 in yeast.

### Functional significance of putative metal-binding domains and metal coordination sites in HvHMA2

We used the yeast system to study the functional significance of key residues in HvHMA2 and the relative importance of particular regions of the protein. The N-terminal domain contains the GxCCxxE motif that appears to be conserved in the N termini of all plant P_1B-2_ ATPases; like the N-terminal GxxCxxC motifs of Cu-transporting ATPases these motifs may bind and also regulate metal transport [34]. Mutation of either of the cysteine residues in AtHMA4 abolished its ability to complement the Zn hypersensitivity of *zrc1* and the Cd hypersensitivity of the *ycf1* mutant yeast strains [26]. Mutagenesis studies indicate the CCxxE motif binds Zn and Cd with greater affinity than other metals, and mutation of these residues alters the metal-binding affinity of the N-terminal domain and reduces the ATPase activity but not the metal dependence of the pump [35]. In addition, the N-terminally deleted AtHMA2 and a mutant in which the cysteine residues in the GICCTSE motif were mutated failed to restore the growth of the Arabidopsis *hma2hma4* mutant to wt levels as seen for plants transformed with a non-mutated version of AtHMA2 [28]. This suggests that the N-terminal region is crucial for function and this is supported by the results shown here for HvHMA2, with deletion of this region eliminating Cd sensitivity conferred to wt yeast and Zn resistance conferred to the *zrc1cot1* mutant. We cannot rule out effects on targeting and expression levels at this stage and further studies are necessary to determine whether it is the vicinal cysteines and the subsequent glutamate which have a crucial role as seen for AtHMA2.

The most marked effect of deleting the C-terminus of HvHMA2 was seen in the *zrc1cot1* yeast mutant as this conferred greater Zn resistance than the full length version. Whether this is due to transport differences, or to targeting differences with more of the mutant form being present at the PM pumping Zn out of the cell is not known. The C-terminal region may also function as an autoregulatory domain as has been suggested for AtHMA4 [15], [30]. The C-terminal domain of AtHMA2 is not essential for function *in planta* as deleting it seemed to have only a minor effect on the ability of this pump to restore the growth of the *hma2hma4* mutant [28]. In contrast, deletion of this domain in AtHMA4 suppressed its rescue of this mutant suggesting an important role *in planta* [13]. Results expressing AtHMA4 constructs in tobacco suggest that the full-length pump is required for enhanced transfer of Zn from root to shoot [36]. Deletion of the rice OsHMA2 C-terminus reduces its ability to translocate Zn and Cd from root to shoot [23]. Expression of the AtHMA4 C-terminal region alone in yeast confers strong Cd resistance to wt yeast [29], [13] and also Zn resistance to the *zrc1cot1* mutant [13]. Studies indicate that this is due to binding of Zn and Cd [30]. In contrast, expression of the C-terminal region from HvHMA2 has little effect on yeast metal tolerance suggesting that this may not show strong binding of these metals in yeast.

Analysis of SERCA pumps and sequence comparisons between different classes of P_1B_-ATPases has allowed key residues to be identified that may be important in metal coordination and transport [8], [18], [37], [38]. We identified invariant residues and key putative metal coordination residues in TMs 6 and 7 of HvHMA2, and tested the effect of these mutations on the yeast Cd response. Following expression in wt yeast, mutants P357L (in the CPC motif), S363L, P365L, K666L and N658L all decreased the Cd sensitivity compared to non-mutated HvHMA2 with P357L and K666L being the most effective. All mutation also reduced Zn resistance conferred by *HvHMA2* to *zrc1cot1*.This suggests that these residues are important and in some cases crucial for transport function, although we cannot rule out effects on expression levels or targeting as being influencing factors. Further studies are now required to determine the exact functional significance of these mutations in HvHMA2 and epitope tagging could help answer the question of localisation. Few studies have tested the functional significance of different residues in plant sequences. Mutating the CPC motif to GPC eliminated the Cd and Zn resistance conferred on yeast by AtHMA4 [15], and CPC to SPC in AtHMA4 abolished its ability to rescue the *ycf1* mutant on elevated Cd and the *zrc1* mutant on high Zn [26]. Interestingly, substitutions in the P_1B-1_ ATPase AtHMA5 of both the latter proline in CPC(x)_6_P motif of TM6 in the Chisdra-2 ecotype and of N923 in the Cape Verde Island ecotype (TM7, equivalent to HvHMA2 N658), were both associated with Cu sensitivity and low capacity of Cu translocation from roots to shoots, indicating that these are also important residues in the P_1B-1_ subclass of pumps [39].

Some of these residues have been investigated in P_1B-2_ ATPases from other organisms. For HvHMA2 we observed the greatest decrease in HvHMA2-conferred Zn resistance with the mutants K666L, P357L (within the CPC motif) and D400A (the phosphorylated aspartate). Similarly in ZntA of *Escherichia coli* the mutation K693N (TM7, equivalent to HvHMA2(K666)) abolished Zn-stimulated ATPase activity completely, although Zn-dependent phosphorylation by ATP still occurred [40]. Also in ZntA, mutants in the CPC motif have been investigated: C392A, P393A, and C394A lost the ability to bind a metal ion with high affinity in the transmembrane domain, while histidine and serine substitutions at C392 and C394 abolished binding of Pb^2+^ but not other divalent metal ions [37]. Our data support a model whereby the CPC motif of TM6 and the conserved lysine in TM7 are parts of the transmembrane metal-binding site.

### HvHMA2 suppresses the stunted phenotype of the *hma2hma4* mutant by partially restoring the elemental balance

Root to shoot Zn transfer is obviously an important step in crops and this would be a key process in barley to ensure that Zn is moved to the shoot where it could be available for transport to the grain; HvHMA2 could potentially play a role in root to shoot transport. The Arabidopsis *hma2hma4* mutant is defective in root to shoot translocation of Zn, and can be rescued by application of high levels of Zn to the soil [12]. This is a useful system for investigating the function of Zn/Cd P_1B_-ATPases and exploring the ability of related pumps to transport Zn [28], [13]. We showed that HvHMA2 can function in root to shoot transfer as when expressed in the *hma2hma4* mutant it suppressed the stunted phenotype, restoring growth to wt levels. HvHMA2 expression also resulted in a small but significant increase in shoot Zn concentration (determined in leaves before bolting). It has previously been noted that partial rescue of the *hma2hma4* shoot Zn concentration to around 30% of wt levels was seen in *AtHMA4*-*hma2hma4* lines [13] and this was sufficient to fully rescue the stunted phenotype of the *hma2hma4* double mutant. A similar rescue is seen here when expressing *HvHMA2* indicating that the barley transporter, HvHMA2 enhances root to shoot transport of Zn when expressed in Arabidopsis.

*HvHMA2*-*hma2hma4* plants also flowered and produced siliques but these were generally shorter than wt. We have previously observed this for *AtHMA4*-*hma2hma4* plants [13]. The only element that was significantly reduced in siliques of *HvHMA2*-expressing lines was Zn (Figure 9), suggesting that the reduced Zn content may lead to the shorter siliques observed here. Indeed reduced silique length has been observed previously in Arabidopsis wt plants when Zn supply was reduced [41]. It would be interesting to express *HvHMA2* under the *AtHMA2* or *AtHMA4* promoter to determine whether the partial rescue of the siliques was a consequence of expression under the 35S promoter rather than a difference in activity of AtHMA2/4 and HvHMA2.

Interestingly, the shoot Cu concentration of the *hma2hma4* mutant is higher than the wt [13]. When *HvHMA2* is expressed in the *hma2hma4* mutant, Cu levels in shoots are reduced towards wt as seen when expressing *AtHMA*4 in this mutant [13]. It could be that this is due to these pumps partially restoring the Zn balance which then has the indirect effect of restoring the Cu balance, as no direct transport of Cu by HvHMA2 or AtHMA4 has been shown.

### Role of HvHMA2 in Zn transport in grain

We analysed *HvHMA2* expression in a number of published microarray datasets. A time course of gene expression in developing barley grain indicated highest expression of *HvHMA2* in ‘endosperm plus aleurone’ at 16 and 25 days after flowering. Expression in these tissues also increased following imbibition (figure S3b). In comparison, expression in ‘embryo plus scutellum’ increased during grain development and was maximal in the mature grain; it decreased during imbibition (figure S3b). Laser capture microdissection was used to study expression of potential Zn transporter genes in the different cell layers of barley grain at 20 days after pollination and results indicated that *HvHMA2* is most highly expressed in transfer cells with lower levels in the aleurone layer, endosperm and embryo [42]. To extend this data we used real-time PCR to investigate expression of HvHMA2 in germinating grain. HvHMA2 expression is slightly reduced in embryo tissue between 2 and 72 hr imbibition while it is increased in the endosperm cells. Interestingly the remaining tissue left after dissecting out the embryo and endosperm shows the highest levels of HvHMA2 expression indicating that HvHMA2 may function in pumping Zn in this tissue to the endosperm and embryo.

In summary, this study provides evidence that HvHMA2 functions in Zn and Cd transport and may play a similar role to AtHMA2 and 4 in Arabidopsis in transferring these ions from roots to shoots. Further work is required to determine the exact physiological role of this family of pumps in the grain and whether the manipulation of expression levels of HvHMA2 in barley can be used to alter Zn content.

## Experimental Procedures

### Plant materials

For growth and elemental analyses of Arabidopsis plants (wt, *hma2hma4* mutants and *HvHMA2*-transformants), plants were grown as described previously [13]. For elemental analysis of leaf material collected before bolting, plants were grown in soil supplemented with sub-toxic concentrations of various elements including 0.09 ppm Cd and were regularly watered with Fe-HBED and 0.25× Hoagland's solution [43]. Grain from *Hordeum vulgare* L. cultivar Golden Promise was heat treated at 45°C for 48 hours and then imbibed on water soaked absorbent paper in sealed petri dishes at 20°C to initiate germination. Grain tissues were separated and used to prepare RNA. To isolate leaf and root material from more mature plants, barley plants were grown on vermiculite.

### DNA and RNA isolation and cDNA synthesis

Genomic DNA was prepared using the DNAmite kit (Microzone Ltd, UK). RNA was prepared using a phenol-SDS extraction and LiCl precipitation method based on [44], except for barely mature root and shoot material which was isolated using TRIzol Reagent (Invitrogen Life Technologies). cDNA was produced using the Superscript III kit (Invitrogen, UK).

### RT-PCR to detect expression of *HvHMA2* in *hma2hma4* mutants

RT-PCR was performed with Biomix taq (Bioline, UK). All primers used in this study are given in Table S3. *Actin 2*, used as the control, was amplified using primers spanning an intron (Actin2.f = 5′-ggtaacattgtgctcagtggtgg-3′, Actin2.R = 5′-ctcggccttggagatccacatc-3′, 28 cycles) while the transgene, *HvHMA2* was detected using primers HvHMA2rt.F (5′-tcaatgcagcacagaacaca-3′) and HvHMA2rt.R (5′-ggccagcttgaacaaacatt-3′) (30 cycles). Real-Time PCR reactions were carried out as previously described [45] using the above primers for *HvHMA2*. RNABP was the control gene with primers RNABP-F (5′- cgcccagttatccatccatcta-3′) and RNABP-R (5′- aaaaacaccacaggaccggac-3′).

### Cloning of HvHMA2 and creation of Entry clones for Gateway-cloning

Partial sequence for *HvHMA2* (from 3^rd^ TM into 3′ UTR) was obtained by alignment of *AtHMA2*, *3* and *4* sequences with barley EST sequences (http://harvest.ucr.edu/, http://www.plantgdb.org/, http://www.scri.ac.uk/, http://earth.lab.nig.ac.jp/ and http://www.ncbi.nlm.nih.gov/) and primers HvHMA2rB, HvHMA2fC(EcoRV), HvHMA2hingeR, and HvHMA2u3R(EcoRI) were designed. *HvHMA2* N-terminal sequence was obtained by 5′ RACE (Generacer kit, Invitrogen) using the RACE forward primer and reverse primer HvHMA2rB on leaf cDNA. The PCR product was re-amplified using the nested RACE forward primer and HvHMA2rB. The resultant sequence was used to design the primer HvHMA2atgF(EcoRV) spanning the HvHMA2 translational start. N-terminal and C-terminal halves of the protein were amplified from barley leaf cDNA using primers HvHMA2atgF(EcoRV) and HvHMA2hingeR or HvHMA2fC(EcoRV) and HvHMA2u3R(EcoRI), respectively. Full length *HvHMA2* was amplified using primers HvHMA2atgF(EcoRV) and HvHMA2u3R(EcoRI) with Pfu DNA polymerase (Promega) (Ta 60°C), then the 3 kb product was re-amplified. 5′ A overhangs were added and the product was AT-cloned into pGEM-T easy (Invitrogen) to create pGEMTe.HvHMA2.FL. Sequencing confirmed EST and RACE data. Full length *HvHMA2* sequence was amplified from this using a topo-adapted forward primer (HvHMA2topoF) and the reverse primer HvHMA2with-stop. The resultant PCR product was topoisomerase-cloned into pENTR/D-TOPO (Invitrogen) and transformed into *E.coli* to create pENTR:HvHMA2(with-stop). The *HvHMA2* insert was fully sequenced.

### Sequence analysis

Hydropathy analysis was performed with Expasy protscale (http://www.expasy.ch/tools/protscale.html).

The sequence alignment was prepared using ClustalW2 [46] and annotated with GeneDoc (www.psc.edu/biomed/genedoc) using information from Swissprot (http://www.expasy.ch/) and SOSUI (http://bp.nuap.nagoya-u.ac.jp/sosui/; [47]. Brachypodium, sorghum and maize sequences from http://aramemnon.uni-koeln.de/ are: Bd1g34140, Bd1g53670, Sb02g006940, Sb02g006950, Sb10g028920, Zm455491, ZmAC205008_FGT002, Zm175576 and Zm099191. NCBI accession numbers are: TaHMA2: DQ490135; OsHMA2 and OsHMA3: ADU53143 and BAJ25745 respectively (both from cv japonica). Barley sequences are BAK06002 (HvHMA1), BAK00726, BAJ93769, BAJ93251, BAK07450, BAJ87066 (HvHMA3 to 7 respectively) and BAJ96159 (HvHMA9). For HvHMA8 and other rice sequence details see [18]. The dendrogram was constructed using ClustalW2 and Treeview [48]. Sequence similarity and identity was calculated using MatGAT 2.02 [49]. Barley and wheat HMA2 expression data were retrieved using Webcomparator (http://contigcomp.acpfg.com.au/) [50].

### Generation of HvHMA2 transformed Arabidopsis plants

HvHMA2 plant expression constructs were created by Gateway (Invitrogen) recombination of pENTR:HvHMA2(with-stop) into pEarleyGate100 [51] to create the non-tagged construct pEG100 35S::HvHMA2, and into pMDC43 [52] to create pMDC43 35S::GFP-HvHMA2 with GFP fused to the N-terminus of HvHMA2. Constructs were electroporated into *Agrobacterium tumefaciens* GV3101 and transformed using the floral dip method [53] into Arabidopsis wt or *hma2hma4* plants (grown with Zn) [12], [13].

### Preparation of HvHMA2 constructs for expression in yeast

*HvHMA2* and mutants (below) were recombined into pYTV [54], under the galactose-inducible Gal1 promoter.

#### Deletion mutants

*HvHMA2* deletion mutations generated by PCR and topo-cloned into pENTR/D-TOPO were: HvHMA2Δ714-1009 (deletion of predicted cytoplasmic C-terminus); primers HvHMA2topoF and HvHMA2Δ714-1009R. HvHMA2Δ2-81 (deletion of predicted cytoplasmic N-terminus); primers HvHMA2Δ2-81F and HvHMA2with-stop. HvHMA2Δ2-698 (deletion of all except predicted cytoplasmic C-terminus); primers HvHMA2Δ2-698F and HvHMA2with-stop.

#### Transport-null substitution mutants HvHMA2(D400A) and HvHMA2Δ714-1009 (D400A)

Site-directed mutagenesis (Quikchange XL, Stratagene) (primers HvHMA2D400A and HvHMA2D400Arc) was performed on pENTR:HvHMA2(with-stop) and pENTR:HvHMA2Δ714-1009 to mutate the critical phosphorylated aspartate of the D_400_KTGT motif to alanine.

#### *HvHMA2* substitution mutants of proposed ion-specificity determinant residues

HvHMA2(P357L), HvHMA2(S363L) and HvHMA2(P365L) were created by PCR with primers HvHMA2Δ2-81F and either HvHMA2-P357L.R, HvHMA2-S363L.R or HvHMA2-P365L.R. Residues were mutated to leucine as this is a hydrophobic amino acid suggested to cause minimal disruption to helix formation while still having a relatively small side chain [55]. Products were AatII/FspI-digested and inserted into AatII/FspI pENTR:HvHMA2(with-stop). Primer HvHMA2-P357L.R also includes a silent mutation which abolishes a SalI site. HvHMA2(N658L) and HvHMA2(K666L) were made similarly with primers HvHMA2fC(EcoRV)and HvHMA2-N658L.R or HvHMA2-K666L.R. The product was FspI/MfeI-digested and inserted into FspI/MfeI pENTR:HvHMA2(with-stop).

### Yeast strains and heterologous expression of various HvHMA2 constructs

Wt *S. cerevisiae* BY4741, the Cd-sensitive *ycf1* mutant, and yeast transformation were as previously described [15], [45], [13]. The Zn-hypersensitive *zrc1cot1* mutant (*MATa;his3*Δ*1;leu2*Δ*0; met15*Δ*0;ura3*Δ*0; zrc1::natMX cot1::kanMX4*) was obtained from Dr. U. Kramer (Heidleberg, Germany).

### Metal sensitivity tests of HvHMA2 transformed yeast

Tests were performed as described previously [13] except plates were incubated for 2 days.

### Microscopy

Seedlings of both 35S::GFP-HMA2 expressing plants and wt Col0 were grown as described in [56]. Plants were mounted on microscope slides in water for imaging using a Zeiss LSM 510META confocal system (Carl Zeiss Ltd., Welwyn Garden City, UK). GFP and chlorophyll were excited using the 488 nm line of an argon ion laser. GFP emission was detected between 505–530 nm and chlorophyll autofluorescence was detected using a LP580 filter.

### Ionomic analysis

Elemental analysis of leaf material before bolting was carried out using ICP-MS [43]. Elemental analysis of silique material was carried out by ICP-OES.

### Accession numbers

The Genbank accession number for *HvHMA2* cDNA cloned in this study is GU177852.

## Supporting Information

Figure S1 **RT-PCR amplification of** ***HvHMA2*** **.** The N-terminal and C-terminal parts and the full length *HvHMA2* sequence were amplified from barley leaf cDNA (lanes 1–3 respectively) using information from EST analysis and 5′ RACE. Full-length *HvHMA2* PCR product was re-amplified from the ∼3 kB product (lane 4).
(TIF)

Figure S2 **Hydropathy analysis of P_1B-2_–ATPases.** Hydropathy analyses indicate that locations of predicted TM domains are highly conserved in the primary structure of P_1B-2_ -ATPases.
(TIF)

Figure S3 **Tissue expression pattern of** ***HvHMA2*** **and** ***TaHMA2*** **.** a. Microarray expression data for barley (solid line) and wheat (broken line) indicates HMA2 is expressed in all tissues, with highest expression in anthers. Unbroken line, HvHMA2; broken line, TaHMA2. Tissue key: gem: germinating seed embryo; rad: germinating seed radicle; roo: germinating seed root; col: germinating seed coleoptile; cro: seedling crown; lea: seedling leaf; brc: floral bracts before anthesis; inf: immature inflorescence; ant: anthers before anthesis; pst: pistil before anthesis; car5: caryopsis 5 DAP (days after pollination); en22: endosperm 22 DAP; em22: embryo 22 DAP; car10: caryopsis 10 DAP; car16: caryopsis 16 DAP. b. Normalized expression values for two replicate experimental series based on independently grown plant material indicates HvHMA2 expression in grain tissues varies during grain maturation and germination. Time: daf, days after flowering; hai, hours after imbibition. Data extracted from supplementary data of [57].
(TIF)

Figure S4 **Tissues used for RT-PCR.** Top: Preparation of tissues for RNA extraction after 2 h imbibition. Bottom: Preparation of tissues for RNA extraction after 72 h imbibition or longer.
(TIF)

Figure S5 **Zn restores growth of the Arabidopsis** ***hma2hma4*** **mutant to wild-type levels.** Top, Rosette diameter measured in plants with or without Zn (3 mM) supplied throughout the growth period. Mean (±S.E) is shown from a representative experiment (n = 12 plants). Bottom, representative plants showing the effect of Zn on the growth of *hma2hma4* mutant.
(TIF)

Figure S6 **Arabidopsis** ***hma2hma4*** **plants are expressing** ***HvHMA2*** **.** RT-PCR shows expression of *HvHMA2* (top) in four independent lines of the Arabidopsis *hma2hma4* mutant transformed with HvHMA2 under the 35S-promoter (35S::HvHMA2 *hma2hma4* lines). Wild-type (wt) and *hma2hma4* mutant are shown as controls. Actin control levels are similar for all lines (bottom).
(TIF)

Table S1 **Analysis of protein sequence homology between HvHMA2 and P_1B-2_ P-types from Arabidopsis, wheat, rice, sorghum and brachypodium.**
(DOC)

Table S2 **nvariant amino acids in TMs 6, 7 and 8 of**I **P_1B-2_ P-type ATPases** **that may be involved in coordinating metals during transport.**
(DOC)

Table S3 **Primers used for the cloning of** ***HvHMA2*** **and the generation of mutants.**
(DOC)
