# Supplementary figures and images for: HvHMA2, a P1B-ATPase from Barley, Is Highly Conserved among Cereals and Functions in Zn and Cd Transport
Source: PLoS One. 2012 Aug 3;7(8):e42640. doi: 10.1371/journal.pone.0042640 (PMC3411818; doi:10.1371/journal.pone.0042640)

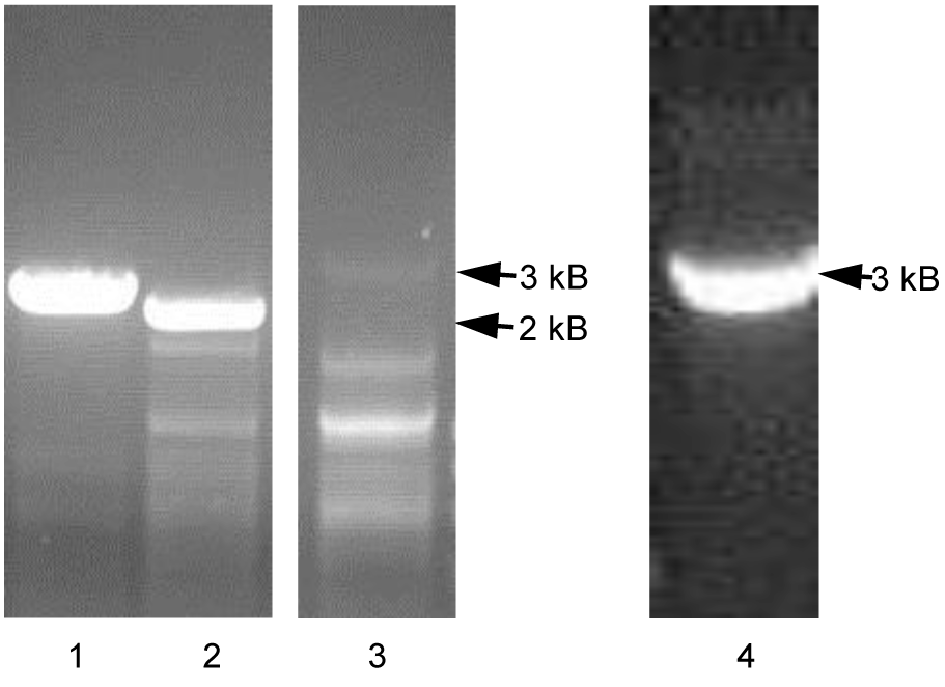

Supplement: Figure S1 — RT-PCR amplification of HvHMA2 . The N-terminal and C-terminal parts and the full length HvHMA2 sequence were amplified from barley leaf cDNA (lanes 1–3 respectively) using information from EST analysis and 5′ RACE. Full-length HvHMA2 PCR product was re-amplified from the ∼3 kB product (lane 4). (TIF) [file pone.0042640.s001.tif]

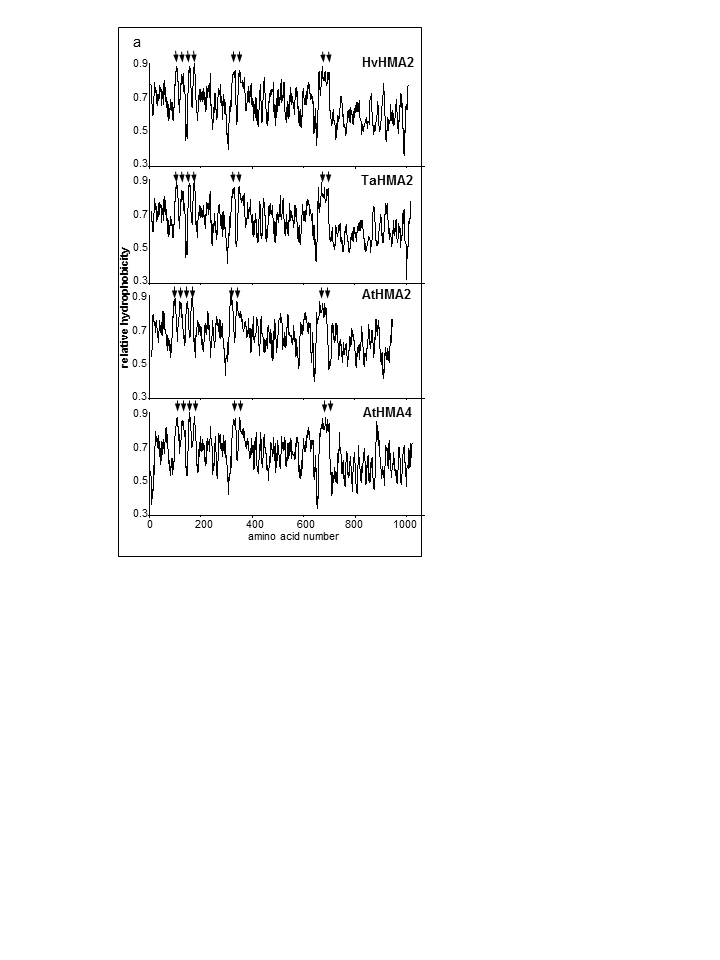

Supplement: Figure S2 — Hydropathy analysis of P1B-2–ATPases. Hydropathy analyses indicate that locations of predicted TM domains are highly conserved in the primary structure of P1B-2 -ATPases. (TIF) [file pone.0042640.s002.tif]

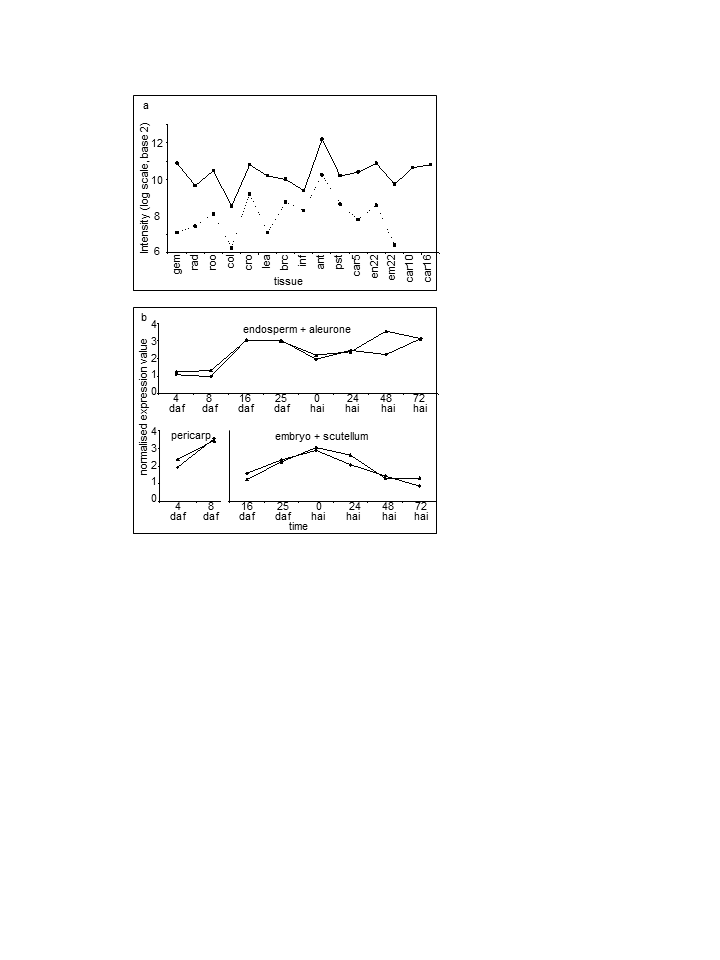

Supplement: Figure S3 — Tissue expression pattern of HvHMA2 and TaHMA2 . a. Microarray expression data for barley (solid line) and wheat (broken line) indicates HMA2 is expressed in all tissues, with highest expression in anthers. Unbroken line, HvHMA2; broken line, TaHMA2. Tissue key: gem: germinating seed embryo; rad: germinating seed radicle; roo: germinating seed root; col: germinating seed coleoptile; cro: seedling crown; lea: seedling leaf; brc: floral bracts before anthesis; inf: immature inflorescence; ant: anthers before anthesis; pst: pistil before anthesis; car5: caryopsis 5 DAP (days after pollination); en22: endosperm 22 DAP; em22: embryo 22 DAP; car10: caryopsis 10 DAP; car16: caryopsis 16 DAP. b. Normalized expression values for two replicate experimental series based on independently grown plant material indicates HvHMA2 expression in grain tissues varies during grain maturation and germination. Time: daf, days after flowering; hai, hours after imbibition. Data extracted from supplementary data of [57]. (TIF) [file pone.0042640.s003.tif]

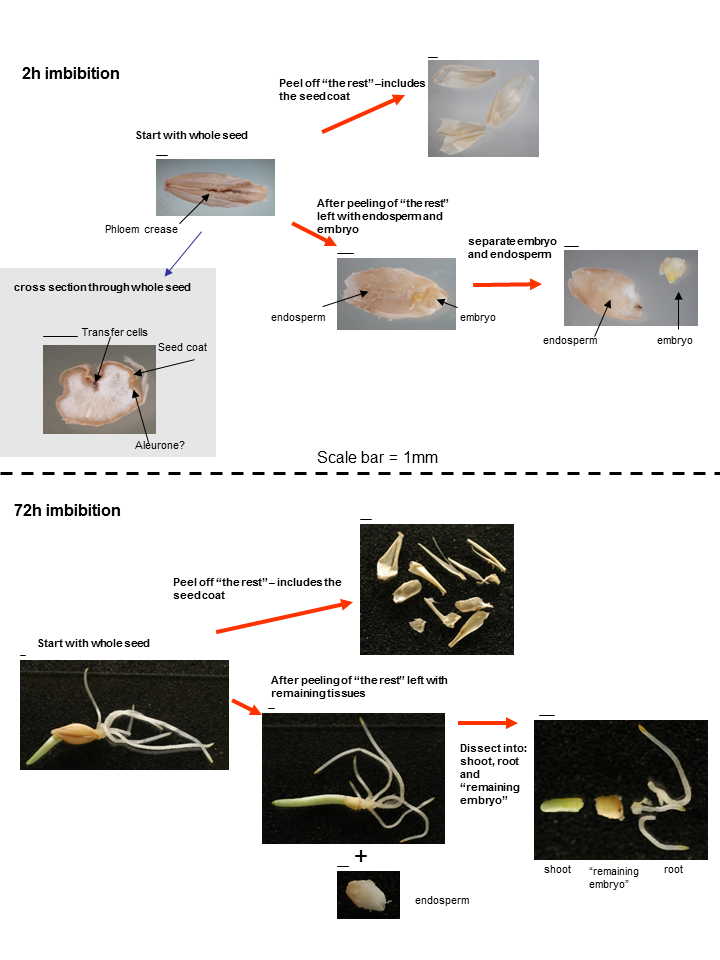

Supplement: Figure S4 — Tissues used for RT-PCR. Top: Preparation of tissues for RNA extraction after 2 h imbibition. Bottom: Preparation of tissues for RNA extraction after 72 h imbibition or longer. (TIF) [file pone.0042640.s004.tif]

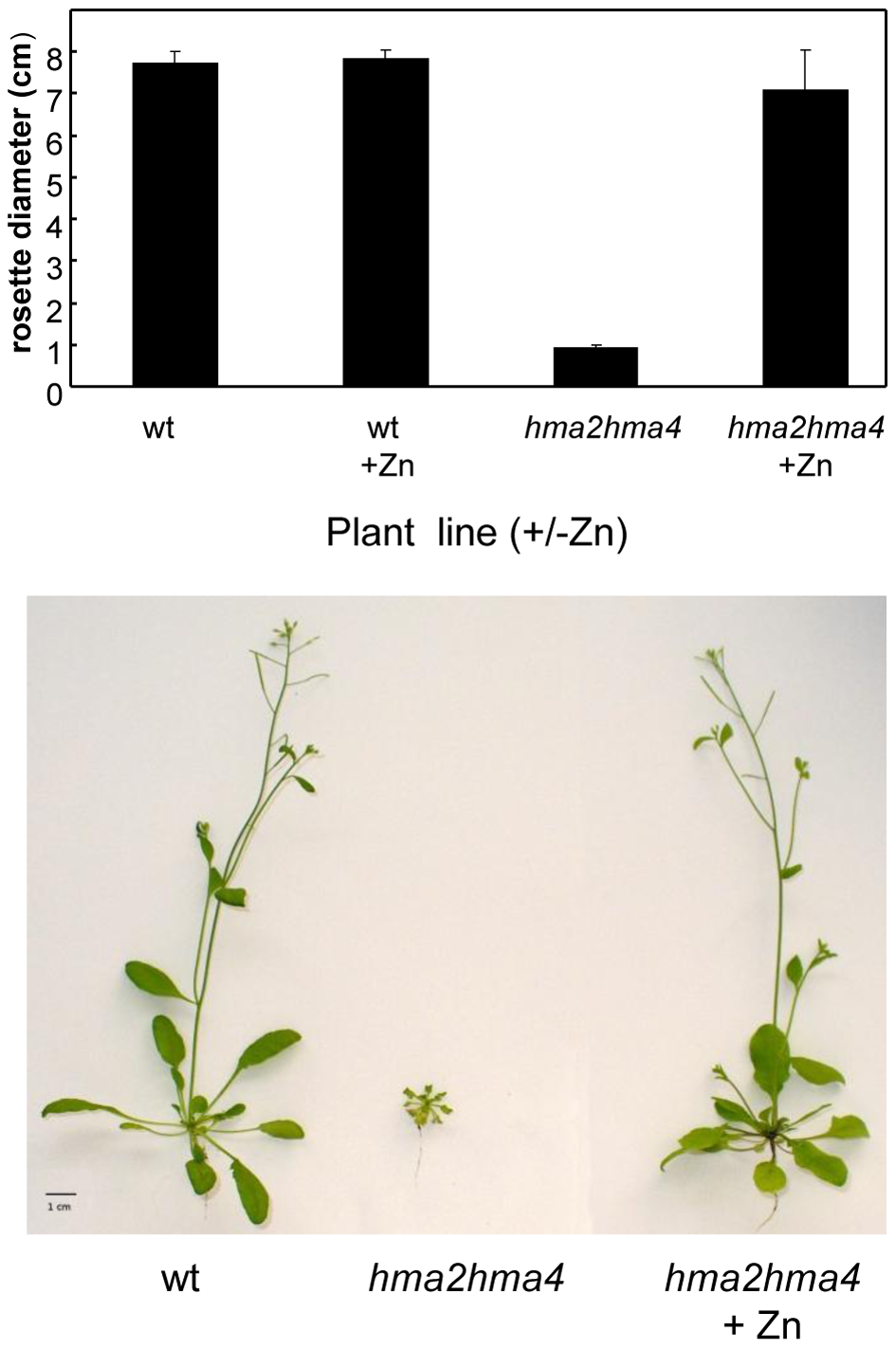

Supplement: Figure S5 — Zn restores growth of the Arabidopsis hma2hma4 mutant to wild-type levels. Top, Rosette diameter measured in plants with or without Zn (3 mM) supplied throughout the growth period. Mean (±S.E) is shown from a representative experiment (n = 12 plants). Bottom, representative plants showing the effect of Zn on the growth of hma2hma4 mutant. (TIF) [file pone.0042640.s005.tif]

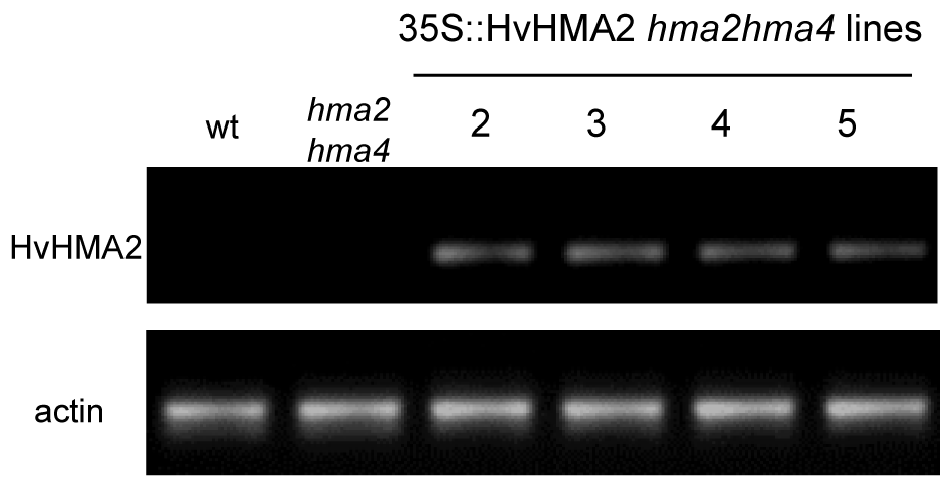

Supplement: Figure S6 — Arabidopsis hma2hma4 plants are expressing HvHMA2 . RT-PCR shows expression of HvHMA2 (top) in four independent lines of the Arabidopsis hma2hma4 mutant transformed with HvHMA2 under the 35S-promoter (35S::HvHMA2 hma2hma4 lines). Wild-type (wt) and hma2hma4 mutant are shown as controls. Actin control levels are similar for all lines (bottom). (TIF) [file pone.0042640.s006.tif]
