# Supplementary material for: HvHMA2, a P1B-ATPase from Barley, Is Highly Conserved among Cereals and Functions in Zn and Cd Transport
Source: PLoS One. 2012 Aug 3;7(8):e42640. doi: 10.1371/journal.pone.0042640 (PMC3411818; doi:10.1371/journal.pone.0042640)
Supplement: Table S1 — Analysis of protein sequence homology between HvHMA2 and P1B-2 P-types from Arabidopsis, wheat, rice, sorghum and brachypodium. (DOC) [file pone.0042640.s007.doc]

Table S1. Analysis of protein sequence homology between HvHMA2 and P_1B-2_ P-types from Arabidopsis, wheat, rice, sorghum and brachypodium.

| Similarity\Identity | Hv  HMA2 | Ta  HMA2 | Bd1g  34140 | Sb10g  028920 | Os  HMA2 | Os  HMA3 | Sb02g  006940 | Sb02g  006950 | At  HMA2 | At  HMA3 | At  HMA4 |
| --- | --- | --- | --- | --- | --- | --- | --- | --- | --- | --- | --- |
| HvHMA2 |  | 91.1 | 81.2 | 69.1 | 71 | 50.1 | 49.5 | 49.8 | 50.2 | 43.1 | 43.2 |
| TaHMA2 | 93.7 |  | 80.5 | 69.9 | 72 | 48.9 | 48.5 | 49 | 49.2 | 42.2 | 43.8 |
| Bd1g34140 | 87.8 | 87.7 |  | 73.1 | 75.5 | 49 | 48.6 | 48.9 | 48.9 | 41.9 | 43.6 |
| Sb10g028920 | 77.7 | 79.4 | 81.7 |  | 74.2 | 48.4 | 46 | 48.2 | 48 | 41.3 | 44.2 |
| OsHMA2 | 80.6 | 81.3 | 84.9 | 83.7 |  | 47.8 | 46.3 | 47.5 | 48.8 | 41 | 44.4 |
| OsHMA3 | 66.5 | 65.1 | 65.6 | 63.8 | 63.7 |  | 62.2 | 57.5 | 44.1 | 39.8 | 39.2 |
| Sb02g006940 | 64.4 | 63.2 | 63.2 | 60.7 | 60.6 | 71.4 |  | 61.8 | 42 | 42.6 | 35 |
| Sb02g006950 | 65.5 | 64.7 | 64.1 | 62.7 | 62.5 | 68.5 | 74.1 |  | 44.3 | 42.9 | 37.1 |
| AtHMA2 | 66 | 65.2 | 64.4 | 62.6 | 64.1 | 62.2 | 61.7 | 62.8 |  | 56.2 | 58.7 |
| AtHMA3 | 57.6 | 57.1 | 56.3 | 54.3 | 54.8 | 54 | 59.3 | 57.8 | 68.8 |  | 45.7 |
| AtHMA4 | 59.9 | 60.2 | 60 | 60.9 | 60.3 | 55.5 | 51 | 53.2 | 68.9 | 56.2 |  |
