# Supplementary material for: HvHMA2, a P1B-ATPase from Barley, Is Highly Conserved among Cereals and Functions in Zn and Cd Transport
Source: PLoS One. 2012 Aug 3;7(8):e42640. doi: 10.1371/journal.pone.0042640 (PMC3411818; doi:10.1371/journal.pone.0042640)
Supplement: Table S2 — nvariant amino acids in TMs 6, 7 and 8 ofI P1B-2 P-type ATPases that may be involved in coordinating metals during transport. (DOC) [file pone.0042640.s008.doc]

Table S2. **Invariant amino acids in TM domains 6, 7 and 8 of** P_1B-2_ P-type ATPases **that may be involved in coordinating metals during transport**

| **Sub-set** | **Amino acid motif** | | |
| --- | --- | --- | --- |
|  | 6^th^ TM | 7^th^ TM | 8^th^ TM |
| IB-2 | CPC(x)_4_SxP | N(x)_7_K | DxG |
