# Supplementary material for: HvHMA2, a P1B-ATPase from Barley, Is Highly Conserved among Cereals and Functions in Zn and Cd Transport
Source: PLoS One. 2012 Aug 3;7(8):e42640. doi: 10.1371/journal.pone.0042640 (PMC3411818; doi:10.1371/journal.pone.0042640)
Supplement: Table S3 — Primers used for the cloning of HvHMA2 and the generation of mutants. (DOC) [file pone.0042640.s009.doc]

Table S3 Primers used for the cloning of *HvHMA2* and the generation of mutants.

| Primer name | Primer |
| --- | --- |
| HvHMA2rB | 5'-TTGATACTGGCCAAGGACTCAAGGAC-3' |
| HvHMA2fC(EcoRV) | 5'-ggatatcgtggccaaaatggcaagg-3' |
| HvHMA2hingeR | 5'-aacgatggtccggtgagtc-3' |
| HvHMA2u3R(EcoRI) | 5'-ggaattcGCTCACTTTGGTTACCGACA-3' |
| HvHMA2atgF(EcoRV) | 5'-ggatatcAGAGAGAGAACGATGGCGGCA-3' |
| HvHMA2topoF | 5'-caccATGGCGGCACCGGCGCCGGCGGCGGCGGGAAA-3' |
| HvHMA2with-stop | 5'-TCATCCTACTATTATCTCAGGTAGTTTCAAC-3' |
| HvHMA2no-stop | 5'-TCCTACTATTATCTCAGGTAGTTTCAACATGC-3' |
| HvHMA2∆714-1009R | 5'-tCATTTCTTCGCCACCTTTCCA-3' |
| HvHMA2∆2-81F | 5'-caccAtggcgtacggcggcgccgggcaga-3' |
| HvHMA2∆2-698F | 5'-caccatggtgctactgagagagaaaggca-3' |
| HvHMA2 D400A | 5'-GCTGCCTTTG**c**CAAGACTGGTACAATTACTAGAGG-3' |
| HvHMA2 D400Arc | 5'-CCTCTAGTAATTGTACCAGTCTTG**g**CAAAGGCAGC-3' |
| HvHMA2fC(EcoRV) | 5'-ggatatcgtggccaaaatggcaagg-3' |
| HvHMA2-P357L.R | 5'-TAGTGCGCAGAAGGTTGCCACGA-3' |
| HvHMA2-S363L.R | 5'-TAGTGCGCAGAAGGTTGCCACGGGTGTCA-3' |
| HvHMA2-P365L.R | 5'-GTGTCGACAGCACCAGAGCACATaGA-3' |
| HvHMA2-N658L.R | 5'-AACAATTGCAAGCTTCGTGGTCACCGAGAAGACAATGAG-3' |
| HvHMA2-K666L.R | 5'-TGCAACAATTGCAAGCAACGT-3' |
